# Successful Treatment of Cyst Infection in an Infant With Autosomal Dominant Polycystic Kidney Disease Using Trimethoprim/Sulfamethoxazole

**DOI:** 10.3389/fped.2020.00216

**Published:** 2020-06-02

**Authors:** Hiroki Yokoyama, Mayumi Sakaguchi, Yuko Yamada, Koichi Kitamoto, Shinichi Okada, Susumu Kanzaki, Noriyuki Namba

**Affiliations:** ^1^Division of Pediatrics and Perinatology, Faculty of Medicine, Tottori University, Yonago, Japan; ^2^Department of Pediatrics, Tottori Prefectural Central Hospital, Tottori, Japan; ^3^Asahigawaso Rehabilitation and Medical Center, Okayama, Japan

**Keywords:** autosomal dominant polycystic kidney disease, cyst infection, trimethoprim/sulfamethoxazole, magnetic resonance imaging, infant

## Abstract

Autosomal dominant polycystic kidney disease (ADPKD) is the most common genetic disease causing renal cysts. Reports on kidney cyst infection in children are rare despite cyst infections being important complications of ADPKD. Here, we report a case of a child without any medical history who had a urinary tract infection with sepsis at 7 months. Leukocyturia persisted despite antibiotic therapy because the infection was treatment-resistant. Initial ultrasound and contrast computed tomography were inconclusive because cysts could not be detected clearly, and a family history of renal cysts was not determined. Subsequently, history of paternal renal cysts, thick walls in infectious cystic lesions on diffusion-weighted magnetic resonance imaging (MRI), and multiple small lesions with high signals on T2-weighted imaging in both kidneys became apparent. Upon diagnosis of ADPKD with cyst infection, antibiotic therapy was switched from cefotaxime to trimethoprim/sulfamethoxazole to achieve better cyst penetration, which successfully resolved the infection. In this patient, MRI was effective for clear visualization and diagnosis of infectious lesions and small cysts in undiagnosed ADPKD with cyst infection. Administering antibiotics with better cyst penetration is important. Trimethoprim/sulfamethoxazole is an option for use in children. This is the first case report that describes ADPKD with cyst infection in an infant in detail.

## Introduction

Autosomal dominant polycystic kidney disease (ADPKD) is the most common inherited cystic kidney disease that affects 1 in 400 to 1,000 individuals. Renal cysts grow and increase bilaterally with age. Approximately half of patients progress to end-stage kidney disease by the age of 60 years (1, 2). Autosomal dominant polycystic kidney disease is believed to be an adult disease; however, recent reports show that symptoms can appear in children and neonates (3–5). Early intervention is beneficial for improving renal prognosis in certain patients. There is increasing awareness on the importance of early diagnosis and treatment.

Autosomal dominant polycystic kidney disease is diagnosed on the basis of family history and presence of cysts on imaging. However, cysts are smaller in children than in adults, making diagnostic imaging less effective. Diagnostic imaging options include ultrasound, computed tomography (CT), and magnetic resonance imaging (MRI) for cysts that are too small for detection by ultrasound.

Cyst infection is a serious complication of ADPKD, which occurs at a rate of 0.01 episodes/patient per year (6). Autosomal dominant polycystic kidney disease cyst infection is rare in children. To the best of our knowledge, there are no case reports in infants. Physiological findings, such as fever, increased inflammatory response, and pain in the site of infection, as well as imaging findings are important for the diagnosis of cyst infection. However, differentiation of cysts and infectious lesions is often difficult by CT or MRI (6, 7). We report the case of a patient who was successfully treated for initially undiagnosed infantile ADPKD with cyst infection.

## Case Presentation

**Figure 1:**
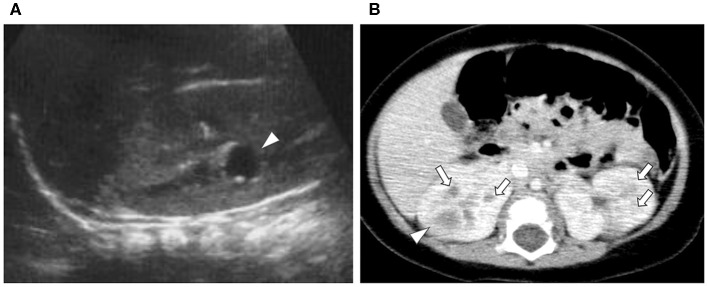
Ultrasound and contrast-enhanced computed tomography (CT). **(A)** Ultrasound at first visit (Day 1): a 1-cm low echo cyst-like region in the right kidney (arrowhead). **(B)** First contrast-enhanced CT (Day 36): the kidney is enlarged. Poorly enhanced masses with enhanced borders were found in both kidneys (arrowhead). Two to three patchy poorly contrasted areas were found in both kidneys, but it was difficult to identify if they were cysts or infectious lesions (arrow).

**Figure 2:**
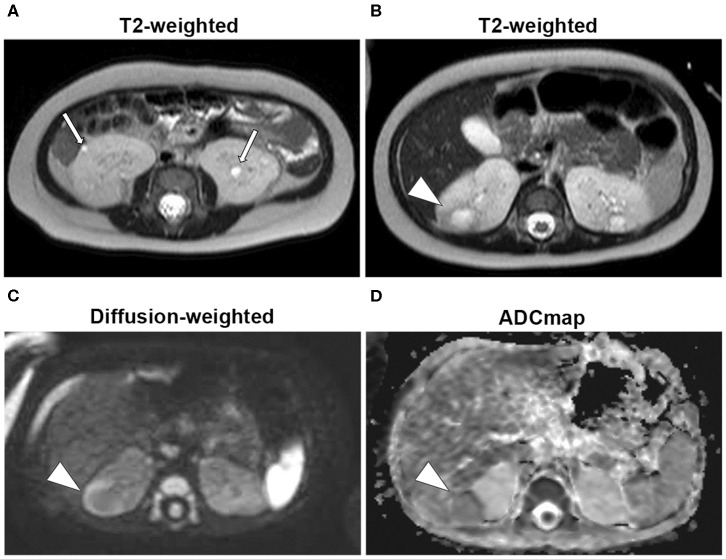
Magnetic resonance imaging (Day 60). **(A)** T2-weighted images: 2-mm high-signal small patchy shadows were found in both kidneys (arrow). **(B)** T2-weighted images: thick walls in the right kidney as low signals and mass as high signals were found in the interior (arrowhead). **(C)** Diffusion-weighted images: masses as high-signal areas of thick-walled structures at the same site as **(B)** (arrowhead). **(D)** Apparent diffusion coefficient (ADC) map: low-signal mass at the same site as **(B)** (arrowhead).

**Figure 3:**
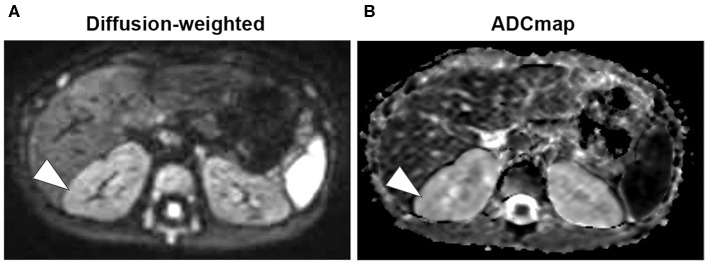
Magnetic resonance imaging (Day 428). **(A)** Diffusion-weighted images showed that the mass with high-signal areas of thick-walled structures disappeared (arrowhead). **(B)** Low-signal masses are also not visible on apparent diffusion coefficient (ADC) map (arrowhead).

**Figure 4:**
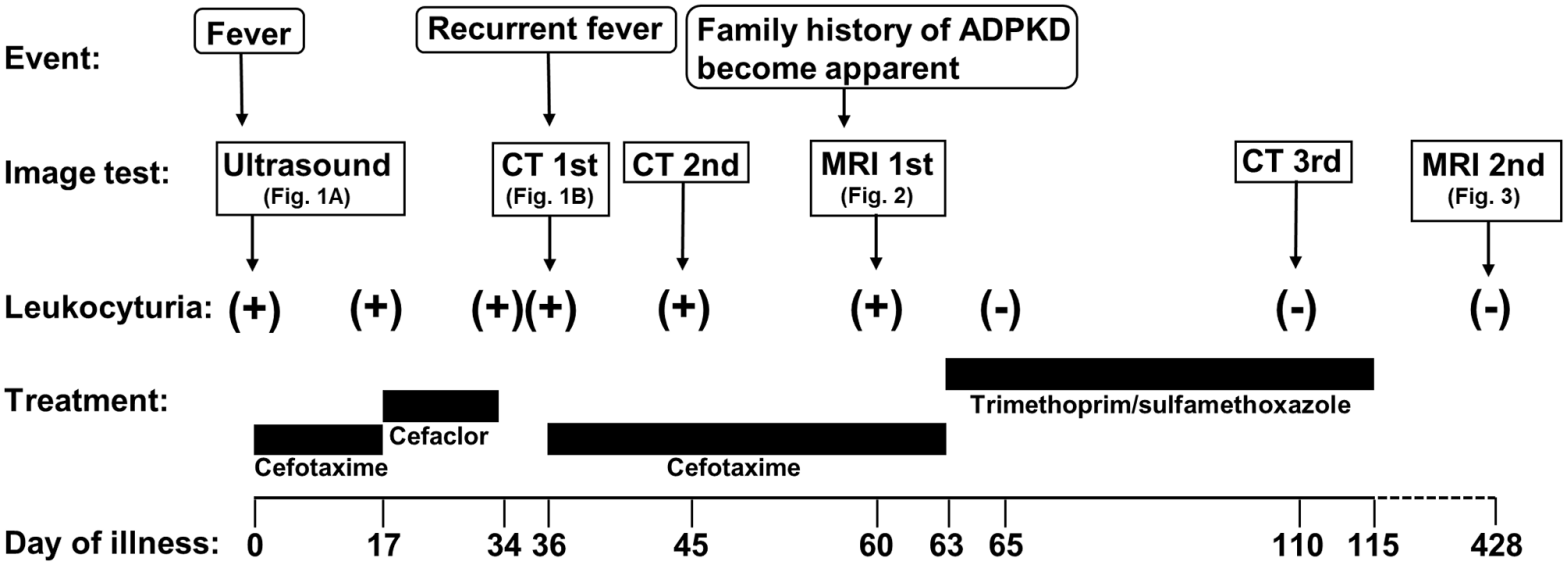
Timeline of the case. ADPKD, autosomal dominant polycystic kidney disease; MRI, magnetic resonance imaging; CT, computed tomography.

A 7-months-old girl with no medical history, and without a family history, presented with fever and fatigue. Her body temperature was 39°C, and blood pressure was 95/55 mm Hg. Blood tests revealed white blood cell count 23.0 × 10^3^/μL, C-reactive protein 4.91 mg/dL, and serum creatinine 0.21 mg/dL. Urine test results revealed positive nitrite reaction and the presence of white blood cells in urine (leukocyturia). Results of urine and blood cultures indicated the presence of *Escherichia coli*; however, cerebrospinal fluid was normal. Detected *E. coli* were not drug-resistant. Ultrasound revealed no specific findings except for a low echoic region in the right kidney, suggesting a simple renal cyst (Figure 1A). Cefotaxime (CTX) therapy (50 mg/kg every 8 h) was initiated for urinary tract infection and sepsis. She became afebrile and stable on day 2 of illness. C-reactive protein increased to 12 mg/dL on the same day, but subsequently decreased and became negative. However, leukocyturia persisted. On day 17, therapy was switched to oral cefaclor (10 mg/kg every 8 h), which was stopped on day 34. On day 36, fever and inflammatory response resumed. Contrast CT showed enlargement of both kidneys and enhanced borders of localized poor contrast areas (Figure 1B); however, there were no findings suggestive of other causes of fever. Blood and urine cultures were negative. Antibiotic therapy (CTX) was reinitiated for suspected renal abscess or acute focal bacterial nephritis (AFBN). Leukocyturia persisted, although the fever promptly abated, and the inflammatory response subsided. Contrast CT was performed again on day 45, but there were no changes in the renal findings of either kidney. By this time, a paternal family history became apparent. The paternal great grandfather and grandfather had history of renal cysts and dialysis, and the paternal great aunt had a kidney transplant. The father was unaware of the family history because his parents had divorced when he was a child. He had no history or subjective symptoms himself at that time. He presented with this opportunity, and multiple renal cysts and liver cysts were observed, then he was diagnosed with ADPKD. Magnetic resonance imaging on day 60 showed more than five small cysts that generated high-signal T2-weighted images for both kidneys (Figure 2A) with low signal appearance T1-weighted images. The right kidney had thick-walled structures on T2-weighted image and diffusion-weighted imaging (DWI), which were visible as a low-signal mass on apparent diffusion coefficient (ADC) map (Figures 2B–D). Based on family history and MRI findings, the patient was diagnosed to have ADPKD with renal cyst infection. Cefotaxime was ineffective; therefore, trimethoprim/sulfamethoxazole (TMP/SMX) therapy [4 mg/kg (trimethoprim component) orally every 12 h] was initiated to achieve better cyst penetration; leukocyturia quickly resolved. Contrast-enhanced CT performed on day 110 showed no enhancement in the borders of the localized poorly contrasted regions, based on which we decided that cyst infection had improved. Trimethoprim/SMX was stopped on day 115 without any side effects during treatment. The patient has had no subsequent recurrence of white blood cells in urine or symptoms of infection. Follow-up MRI at 1½ years showed that the thick-walled structures with high signals on DWI had disappeared (Figure 3). Kidneys were mildly swollen, and multiple cysts were observed on T2-weighted images of both kidneys. The timeline of this case is shown in Figure 4.

## Discussion

In this study, MRI was effective for diagnosing ADPKD incidentally in a patient with undiagnosed cyst infection. The cyst infection was CTX-resistant, but responded promptly to TMP/SMX, which was selected for its more effective delivery to the cyst.

In adults, ADPKD can be diagnosed relatively easily by family history and presence of multiple cysts on imaging tests (8). However, the cysts are small in children and may not be seen on imaging, making the diagnosis more difficult. In adults, ADPKD is diagnosed using ultrasound with >90% accuracy; however, false negatives occur in 38% of children 5 years or younger (8, 9). Both CT and MRI permit detection of cysts smaller than those detectable by ultrasound. In particular, MRI allows detection of cysts ≥2 mm in diameter as high-signal areas on T2-weighted images (10). Diagnostic criteria, including imaging, for ADPKD in infants have not been completely established. Pei et al. (8) suggested that more than 10 cysts in total with family history can be used as a cutoff for making a diagnosis via MRI. But this is applicable for all individuals older than 15 years. In this case, we clinically diagnosed the patient as ADPKD along with the following diagnostic criteria of ADPKD in our region: with family history and five or more bilaterally manifested cysts confirmed on CT and MRI imaging regardless of age (11).

In ADPKD, the most sensitive diagnostic method comprises genetic test (mutated genes PKD1 or PKD2). However, definitive disease-associated mutations are found in only 40–60% cases; therefore, genetic test is used only when other clinical data are questionably supportive of the diagnosis (3). In this case, genetic test was not performed because diagnosis was made based on the family history and MRI scans, and parental consent was not obtained for genetic test.

Furthermore, diagnosing cyst infection in ADPKD is often difficult. Definitive diagnosis of cyst infection is made by cyst puncture to test for infection. Without puncture, it is diagnosed on the basis of combination of imaging and physiological findings such as fever, inflammatory response, and pain in the infected site (7). Diagnostic imaging of cyst infection is also difficult in adults. Differentiating acute pyelonephritis, AFBN, and organic changes secondary to infectious or non-infectious cysts can be problematic even with use of contrast-enhanced CT. A report on diagnostic imaging of cyst infection in ADPKD by ultrasonography, CT, MRI, and positron emission tomography (PET)/CT revealed sensitivities of 6, 25, 71.4, and 95%, respectively (12). Positron emission tomography/CT is reported to be superior in imaging and diagnosing cyst infections (6); however, it is expensive, and radiation exposure is an issue for pediatric patients. In MRI DWI, high signals on DWI, thick walls, and low signals on ADC map are reportedly effective for diagnosing cyst infections (7). The non-inferiority of MRI diagnosis to PET/CT has also been reported. This patient was not initially diagnosed with ADPKD as family history was not known at the time. A differential diagnosis with cysts from renal abscess or bacterial focal nephritis was difficult with ultrasound or CT. In this case, cysts in the infectious lesions were characterized by high signals on DWI with thick walls and low signals on ADC map. Multiple small cysts ≥2 mm in diameter, which were sufficient to diagnose ADPKD, were visible in non-infectious lesions as high signals on T2-weighted images.

Autosomal dominant polycystic kidney disease is highly penetrant, and it is relatively easy to diagnose it based on family history. However, Iliuta et al. (13) reported that ~27.8% of patients with ADPKD had no known family history at their diagnoses owing to the lack of information related to family history in 10.5% and *de novo* disease in 15.3%. In cases of suspected ADPKD, it is important to make a differential diagnosis with the awareness that ADPKD cannot be ruled out despite lack of known family history and to more thoroughly investigate family history.

Fever and the inflammatory response decreased when CTX was administered to the patient at onset. Urine cultures were all negative with the exception of the first. However, once antibiotic therapy was completed, fever and inflammatory response returned, white blood cells in urine persisted, and there were no improvements on CT imaging. Assuming a persistent cyst infection, the patient was administered and responded well to TMP/SMX; it was administered for its superior delivery to the cyst (14, 15). In pathological investigations, cysts of advanced ADPKD have been found to have closed sacs with poor communication with the glomerular filtration system (16). Cyst penetration of antibiotics was reported to be better with liposoluble antibiotics and poorer with hydrosoluble antibiotics. Furthermore, cyst infection in ADPKD develops secondary to pathogens, which are often *E. coli* (75%) and other gram-negative bacilli, introduced via the bloodstream or by traveling up the urinary tract (17). After assessing the results of bacterial cultures, liposoluble and highly cyst-penetrating new quinolones or TMP/SMX are recommended for urinary tract infection in ADPKD. Considering the safety concerns and risk of adverse events of using new quinolones on infants, TMP/SMX was administered for this patient.

Cyst puncture was never performed on this patient; therefore, cyst infection has not technically been proven. However, we diagnosed cyst infection based on the changes on imaging before and after the treatment and the fact that treatment with TMP/SMX was effective. The only persistent symptom during CTX therapy was leukocyturia; this could be because CTX is effective in treating infection in non-cystic tissues.

In conclusion, MRI was effective for diagnosing ADPKD complicated with cyst infection in an infant. The infection was resistant to CTX antibiotic therapy, whereas it was effectively treated with TMP/SMX subsequent to its good delivery to cysts. Magnetic resonance imaging could be effective for differentiating ADPKD and cyst infection in children. Antibiotic therapy, considering cyst delivery, is important in treating pediatric ADPKD cyst infection, and TMP/SMX is a good alternative.

## Data Availability Statement

All datasets generated for this study are included in the article/supplementary material.

## Ethics Statement

Written informed consent was obtained from patient's parents for publication of this case report and any potentially identifying information.

## Author Contributions

HY drafted the initial manuscript and approved the final manuscript as submitted. MS and YY treated and diagnosed the patient. KK, SO, SK, and NN interpreted the patient data and critically revised the manuscript.

## Conflict of Interest

The authors declare that the research was conducted in the absence of any commercial or financial relationships that could be construed as a potential conflict of interest.
